# Transcriptional Profile of Cytokines, Regulatory Mediators and TLR in Mesenchymal Stromal Cells after Inflammatory Signaling and Cell-Passaging

**DOI:** 10.3390/ijms22147309

**Published:** 2021-07-07

**Authors:** Makram Merimi, Karolien Buyl, Dhouha Daassi, Robim M. Rodrigues, Rahma Melki, Philippe Lewalle, Tamara Vanhaecke, Hassan Fahmi, Vera Rogiers, Laurence Lagneaux, Joery De Kock, Mehdi Najar

**Affiliations:** 1 Department of Hematology, Laboratory of Experimental Hematology, Institut Jules Bordet, Université Libre de Bruxelles (ULB), 1000 Bruxelles, Belgium; makram.merimi.cri@gmail.com (M.M.); philippe.lewalle@bordet.be (P.L.); 2 Genetics and Immune Cell Therapy Unit, Faculty of Sciences, University Mohammed Premier, Oujda 60000, Morocco; r.melki@ump.ac.ma; 3 Department of In Vitro Toxicology and Dermato-Cosmetology (IVTD), Faculty of Medicine and Pharmacy, Vrije Universiteit Brussel (VUB), Laarbeeklaan 103, 1090 Brussels, Belgium; Karolien.Buyl@vub.be (K.B.); rmarceli@vub.ac.be (R.M.R.); Tamara.Vanhaecke@vub.be (T.V.); vrogiers@vub.ac.be (V.R.); Joery.De.Kock@vub.be (J.D.K.); 4 Department of Neurology, Icahn School of Medicine at Mount Sinai, New York, NY 10029-5674, USA; bioengdhouha@gmail.com; 5 Osteoarthritis Research Unit, Centre Hospitalier de l’Université de Montréal, University of Montreal Hospital Research Center (CRCHUM), Montreal, QC H2X 0A9, Canada; h.fahmi@umontreal.ca (H.F.); 6 Laboratory of Clinical Cell Therapy, Institut Jules Bordet, Université Libre de Bruxelles (ULB), 1070 Brussels, Belgium; laurence.lagneaux@bordet.be

**Keywords:** mesenchymal stromal cells, transcriptional profile, inflammation, cell-passage, TLR, cytokines, regulatory mediators

## Abstract

Adult human subcutaneous adipose tissue (AT) harbors a rich population of mesenchymal stromal cells (MSCs) that are of interest for tissue repair. For this purpose, it is of utmost importance to determine the response of AT-MSCs to proliferative and inflammatory signals within the damaged tissue. We have characterized the transcriptional profile of cytokines, regulatory mediators and Toll-like receptors (TLR) relevant to the response of MSCs. AT-MSCs constitutively present a distinct profile for each gene and differentially responded to inflammation and cell-passaging. Inflammation leads to an upregulation of IL-6, IL-8, IL-1β, TNFα and CCL5 cytokine expression. Inflammation and cell-passaging increased the expression of HGF, IDO1, PTGS1, PTGS2 and TGFβ. The expression of the TLR pattern was differentially modulated with TLR 1, 2, 3, 4, 9 and 10 being increased, whereas TLR 5 and 6 downregulated. Functional enrichment analysis demonstrated a complex interplay between cytokines, TLR and regulatory mediators central for tissue repair. This profiling highlights that following a combination of inflammatory and proliferative signals, the sensitivity and responsive capacity of AT-MSCs may be significantly modified. Understanding these transcriptional changes may help the development of novel therapeutic approaches.

## 1. Introduction

Mesenchymal Stromal Cells (MSCs) have been shown to be a promising candidate for cell-based therapy [1]. They are relevant for tissue repair and wound healing because of their therapeutic properties. MSCs harbor specific functions such as immunomodulation, trophic support and in vitro differentiation ability into certain connective tissue cells upon specific inductive conditions [2]. Their therapeutic effects are mainly based on their secretome that include a variety of biologically active molecules such as chemokines, cytokines and other regulatory factors. These mediators may modulate diverse biological processes such tissue repair and regeneration, cell-progenitor differentiation and immune/inflammatory responses [3]. Studies have reported that MSCs can migrate and home to specific sites of injury and damage. Stromal cells, inflammation and immune cells are a critical component of the injured environment [4]. There are close interactions between MSCs and tissue local cells such as progenitors and immune cells. MSCs can interact with immune cells from both innate and adaptive immunity. MSCs may induce functional changes of monocytes/macrophages, dendritic cells, T cells, B cells and natural killer cells to finally regulate the immune response [5].

MSCs have been isolated from different tissues including bone marrow, skin, dental pulp and umbilical cord. Due to several concerns with the previous mentioned sources, adipose tissue has been proposed as a valuable alternative for MSCs [6]. Indeed, it is ubiquitously available, can be easily collected with minimal invasive procedure and most importantly harbor a high frequency of MSCs. Our laboratory has gained expertise in isolating MSCs from adipose tissue as well as in characterizing their features. According to the ISCT guidelines [7], MSCs are plastic-adherent in standard cell-culture conditions and display a special immunophenotype and differentiate to osteoblasts and adipocytes in vitro. Thus, MSCs have to present mesenchymal/stromal markers and lack expression of hematopoietic/immune markers. Despite sharing a same phenotype, the secretome of MSCs appears to vary depending on the source and cell-culture conditions of MSCs [8]. Functionally, MSCs are referred as sentinel and therapeutic responsive cells. Indeed, they are highly sensitive and can detect, through a large panel of receptors, stiffness of their environment. For instance, pathogens invading the injured tissue are recognized through pattern recognition receptors (PRRs) such as Toll-like receptors (TLRs). These receptors are expressed by various immune and non-immune cells and mediate signaling required to eliminate harmful stimuli and participate to tissue repair [9]. During the process of wound healing, MSCs may face inflammatory, proliferative and remodeling overlapping signals [10]. MSCs may also be potentially exposed to TLR ligands, which may result in the modulation of their activity and therapeutic efficiency [11,12]. In consequence, MSCs should respond adequately by expressing site-specific mediators that converge to establish a pro-regenerative environment [13].

Manufacturing of MSCs for therapeutic purposes often includes extended cell-expansion or inflammatory licensing. Although these cytokines, chemokines, regulatory mediators and TLR candidate genes are known [14], their profile and modulation under the pressures of cell-expansion and immune activation combination has not been formally investigated. Herein, we have investigated the impact of a combination of inflammation and cell-expansion signals on the transcript profile of genes linked to the immuno-reparative features of AT-MSCs. We have isolated MSCs from adipose tissue by means of the Ficoll gradient centrifugation method and performed several cell-passaging in the presence of inflammation. We found that the expression of cytokines/chemokines (IL-6, IL-8, IL-1β, TNFα and CCL5), regulatory mediators (HGF, IDO1, PTGS1, PTGS2 and TGFβ) and TLR was constitutively different. Of importance, inflammatory signaling and cell-passaging have significantly and differentially modulated these expression profile linked to tissue repair and regeneration. Upregulations of cytokines, regulatory mediator and TLR expression were highlighted demonstrating the great capacity of MSCs to sense and actively respond to tissue challenges. The complex interplay between MSCs, cytokines, TLR and regulatory mediators is central to the process of tissue repair. Moreover, functional enrichment analysis demonstrated that several biological process, molecular functions and cellular components linked to several TLR interaction networks are involved in inflammatory and immune response. Our findings will improve our knowledge regarding the influence of inflammation and cell-expansion on the immune-biology of AT-MSCs, as well as help to identify molecular targets that improve the therapeutic potential of MSCs.

## 2. Results

### 2.1. Source of MSCs

Cells from adipose tissue were isolated by means of Ficoll gradient centrifugation and cultivated by the classical attachment culture method.

### 2.2. The Culture Characteristics of MSCs

**Figure 1:**
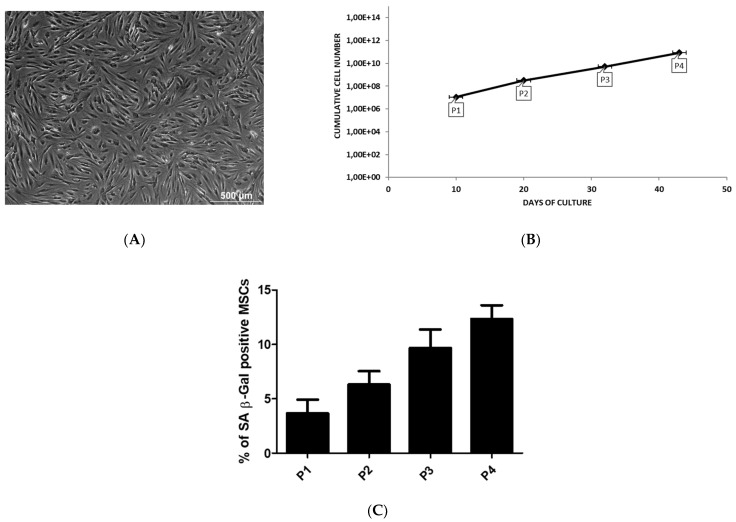
The culture characteristics of adipose MSCs. MSCs are derived from liposuction waste material. The cells are obtained by Gradient Ficoll Centrifugation and cultured by classical adherent method. (**A**) The image is representative for the morphology of MSCs. The cells are observed by light microscopy. Scale bar = 500 µm. (**B**) The cumulative cell number of MSCs at each passage as a function of culture time. (**C**) The percentage of SA β-Gal positive MSCs during passage expansion.

In culture, we observed a heterogeneous population of fibroblastic-shaped cells highly adherent to plastic (Figure 1A).

Cell expansion in culture by cell-passaging allowed to increase the number of MSCs as function of time. Passage 3 and 4 are reached after 32 (5.23E + 09 cells) and 43 (8.62E + 10 cells) days, respectively, with a constant and significant increase in the number of MSCs (Figure 1B). The transcriptional profile of AT-MSCs for cytokines, regulatory mediators and TLR was performed during early (P1–P2) and late (P3–P4) passage that are reached after 15 and 37.5 days, respectively.

In our culture, the level of senescence was very low during the expansion of MSCs and no significant changes was observed between the passages (Figure 1C).

### 2.3. Phenotype of MSCs

MSCs from adipose tissue constitutively expressed the CD105, CD73 and CD90 markers whereas the expression of the CD34, CD45, CD14, CD19 and HLA-DR was negative.

**Figure 2:**
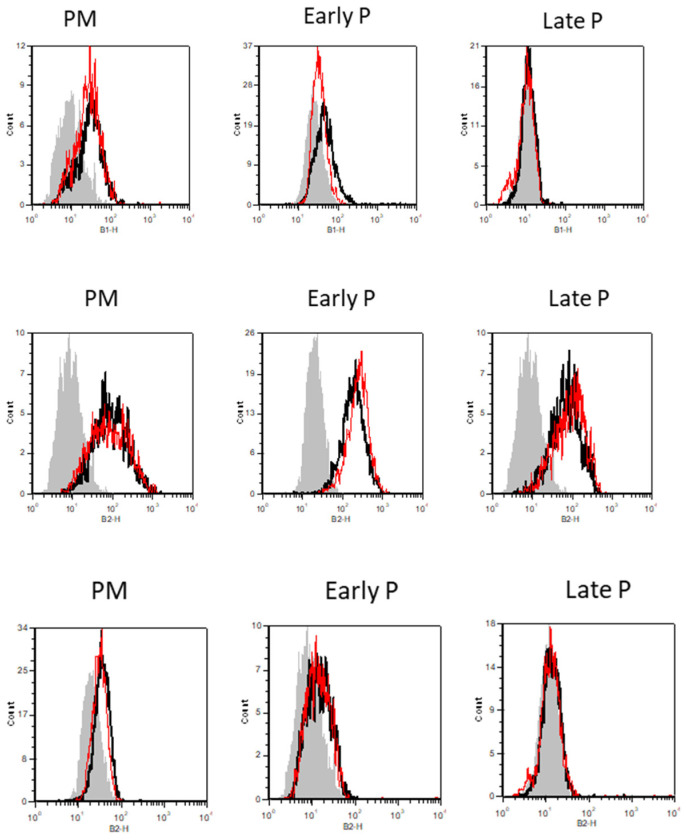

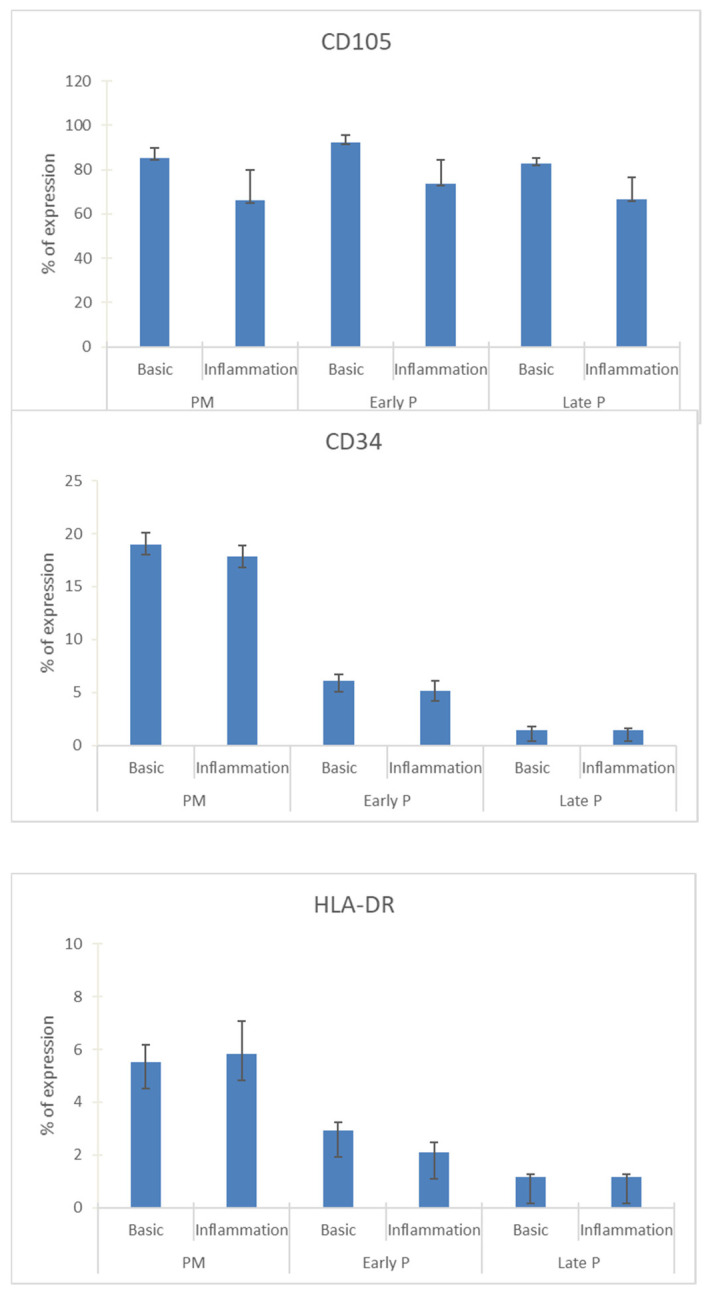
The expression profile of CD34, CD105 and HLA-Dr during cell expansion of MSCs. These markers were analyzed by flow cytometry. The results are presented in the graphic as the mean ± SEM percentage of each marker expression. Representative FACS histograms are also shown for basic (black curve) and inflammation conditions (red curve) during PM, early and late passage cultures. The grey curve represents the antibody control.

Specifically, the % of CD34 expression was in primo culture (PM) 19 ± 1.06 versus 17.83 ± 1.08, in early P 6.08 ± 0.605 versus 5.165 ± 0.915 and in late P 1.42 ± 0.33 versus 1.415 ± 0.2 for basic and inflammation condition, respectively. The % of CD105 expression was in PM 85.33 ± 4.39 versus 66 ± 13.89, in early P 92.33 ± 3.125 versus 73.75 ± 10.525 and in late P 82.75 ± 2.51 versus 66.58 ± 10.155 for basic and inflammation condition, respectively. The % of HLA-Dr expression was in PM 5.5 ± 0.67 versus 5.83 ± 1.25, in early P 2.915 ± 0.32 versus 2.085 ± 0.38 and in late P 1.165 ± 0.105 versus 1.165 ± 0.105 for basic and inflammation condition, respectively (Figure 2).

### 2.4. Differentiation Potential of MSCs

**Figure 3:**
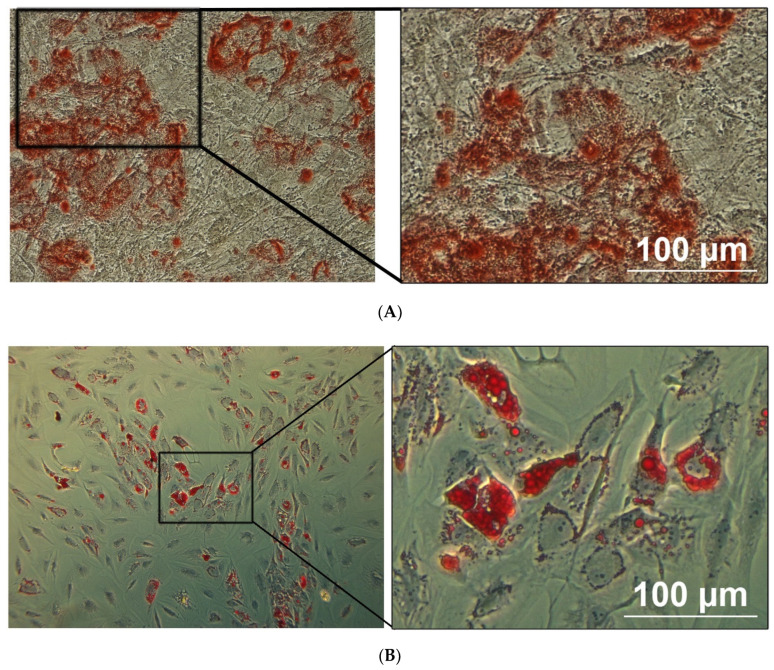
The differentiation potential of adipose MSCs is confirmed by light microscopy. The osteogenic and adipogenic differentiation of MSCs are carried out in specific induction media and confirmed by using lineage-staining techniques. The images are representative for MSCs differentiated into osteoblast (**A**) and adipocytes (**B**), being confirmed by using Alizarin Red (calcium deposits) and Oil red O (Lipid vacuoles) staining, respectively. Scale bar = 100 µm.

Osteogenesis: In contrast to cells cultivated under control conditions, osteoblasts could be detected after 3 weeks of osteogenic induction as shown by the mineral deposition supported by Alizarin Red S staining (Figure 3A). Adipogenesis: In contrast to cells cultivated under control conditions, adipocytes could be detected after 2 weeks of adipogenic induction, as shown by the accumulation of lipid droplets revealed by Oil Red O staining (Figure 3B).

### 2.5. Upregulation of the Cytokine Expression Pattern of MSCs

**Figure 4:**
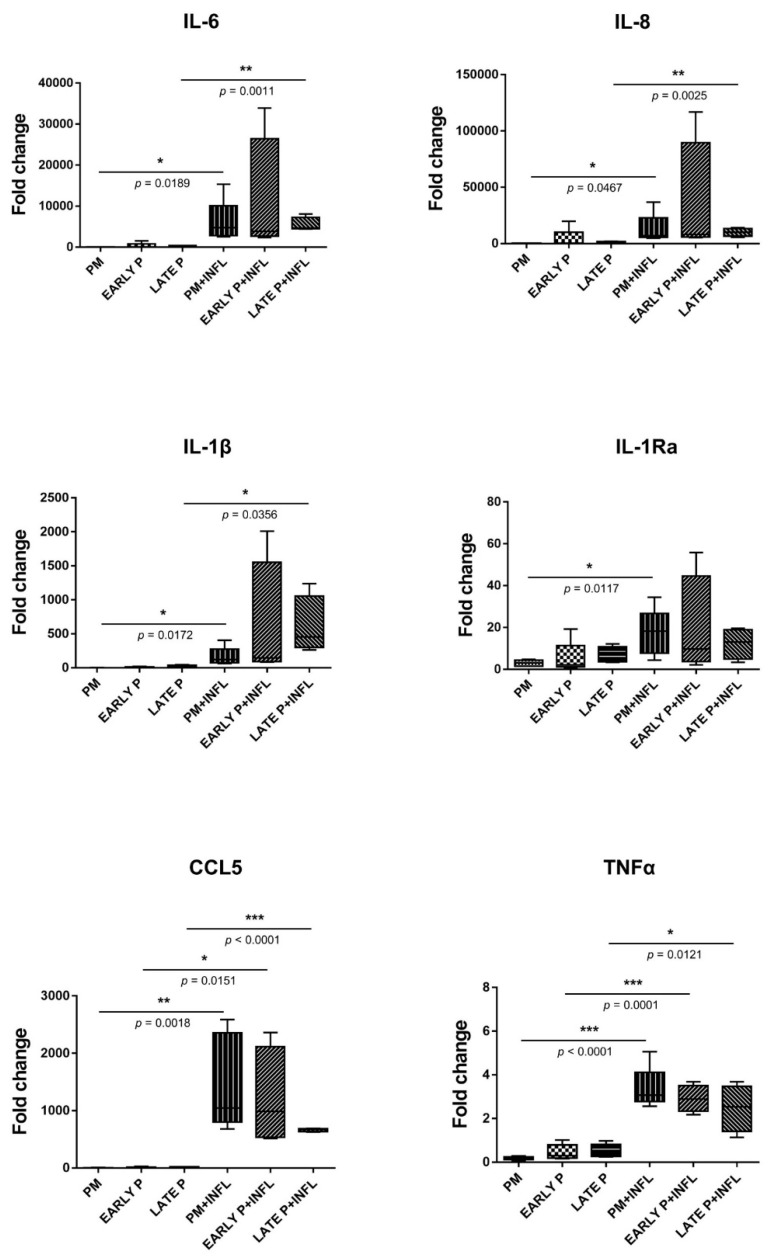
The expression of cytokines is upregulated in MSCs. The gene expression level of each cytokine is determined by qPCR analysis from basic MSCs and inflamed MSCs (INFL) during the primo culture (PM), early (EARLY) and late (LATE) passage (P). The values are expressed as mean ± SEM compared to the expression of the housekeeping gene (ACTB and GAPDH). *, **, *** Significant increase of expression of inflamed MSCs (INFL) versus basic MSCs (*p*-value: *p* ≤ 0.05, *p* ≤ 0.01, *p* ≤ 0.001, respectively).

MSCs constitutively presented a panel of cytokines with different levels of expression (Figure 4). MSCs express interleukin (IL)-6 and IL-8 at high levels, while a lower expression is observed for the chemokine ligand CCL5, IL-1Ra and IL-1β. In contrast, almost no expression of tumor necrosis factor (TNFα) was detected in MSCs. The presence of inflammatory stimulation leads to an upregulation of all cytokine expression regardless of their culture expansion condition. After inflammatory stimulation, IL-6, IL-8, IL-1β, IL-1Ra, TNFα and CCL5 are expressed at higher levels compared to control condition. More specifically, IL-6, IL-8 and IL-1β are significantly upregulated in the primo culture (99.8-fold; 159.2-fold; 130.9-fold, respectively) and late passage (P3-P4) (23.0-fold; 9.8-fold; 41.0-fold, respectively) condition. The same is observed for TNFα (18.0-fold for PM and 5.1-fold for late P) and CCL5 (377.9-fold for PM and 50.6-fold for late P) and on top these cytokines show also a significantly higher expression in the early passage (P1–P2) (7.2-fold and 151.8-fold, respectively) condition. For IL-1Ra, a significantly higher expression (5.8-fold) is observed for the PM condition after pro-inflammatory stimulation compared to the non-inflammatory situation. Further, the cell-passaging is likely to not influence the expression of all these cytokines. More specifically, no significant differences were observed between the primo culture condition, the early (P1–P2) passage condition and the late (P3–P4) passage condition.

### 2.6. Upregulation of the Regulatory Mediator Expression Pattern of MSCs

**Figure 5:**
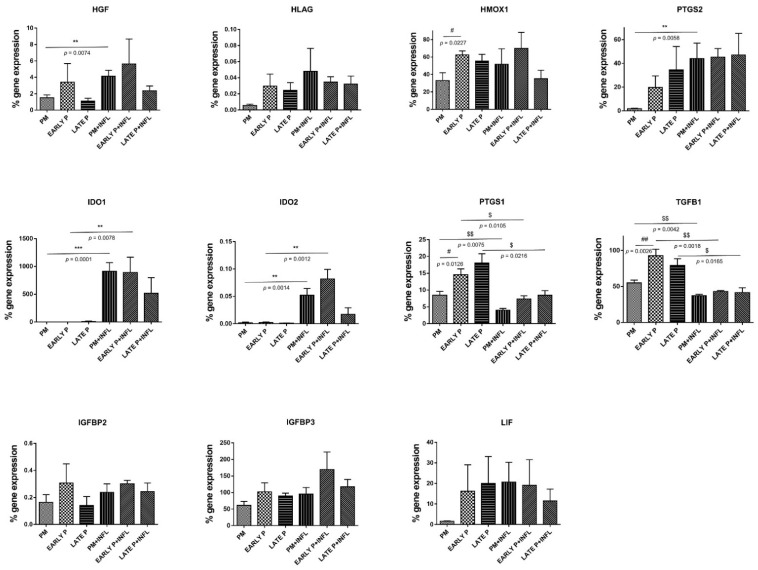
The expression of regulatory mediators is upregulated in MSCs. The gene expression level of each cytokine is determined by qPCR analysis from basic MSCs and inflamed MSCs (INFL) during the primo culture (PM), early (EARLY) and late (LATE) passage (P). The values are expressed as mean ± SEM compared to the expression of the housekeeping gene (ACTB and GAPDH). **, *** Significant increase of expression of inflamed MSCs (INFL) versus basic MSCs (*p*-value: *p* ≤ 0.01, *p* ≤ 0.001, respectively). ^$, $$,^ Significant decrease of expression of inflamed MSCs (INFL) versus basic MSCs (*p*-value: *p* ≤ 0.05, *p* ≤ 0.01, respectively). ^#, ##^ Significantly lower expression compared to the consecutive passage (*p*-value: *p* ≤ 0.05, *p* ≤ 0.01, respectively).

MSCs constitutively presented a panel of regulatory mediators with different levels of expression (Figure 5). MSCs express moderate level of heme oxygenase (HMOX)-1, insulin-like growth factor binding-protein (IGFBP)3 and transforming growth factor (TGF)-β1. Leukemia inhibitory factor (LIF), prostaglandin endoperoxide synthase (PTGS)1 and PTGS2 are expressed at low levels, whilst the expression of hepatocyte growth factor (HGF), indoleamine 2,3-dioxygenase (IDO)1, IDO2, IGFBP2 and HLA-G is insignificant. Both inflammatory signaling and cell-passaging differentially modulated these expression profiles. No significant differences are detected for the expression of HLA-G, IGFBP2, IGFBP3 and LIF despite the cell-passaging or inflammatory setting. Of note, the level of IGFBP3 expression was substantially more elevated than that of IGFBP2. Although, no significant differences are observed for the expression of HGF, IDO1, IDO2 and PTGS2 during the cell-passaging, a significant effect of inflammation is noted. On the other hand, a significant lower expression is perceived for HMOX1 (33.16% ± 8.92% to 62.41% ± 4.49%), PTGS1 (8.45% ± 1.10% to 14.59% ± 1.70%) and TGFβ1 (54.94% ± 3.93% to 92.84% ± 8.99%) in the PM compared to the early passage condition. When comparing the inflammatory and non-inflammatory groups, a significant higher expression is observed for IDO1 and IDO2 in the PM (913.10% ± 154.30% to 0.03% ± 0.01% and 0.05% ± 0.01% to 0.00% ± 0.00%, respectively) and early passage (890.30% ± 275.30% to 0.14% ± 0.12% and 0.08% ± 0.02% to 0.00% ± 0.00%, respectively) condition. In contrast, PTGS1 displayed a significant lower expression for PM (4.03% ± 0.51% to 8.50% ± 1.10%) and early passage (7.38% ± 0.89% to 14.59% ± 1.70%) and on top a significant difference is detected for the late passage (8.48% ± 1.36% to 18.04% ± 2.78%) condition after inflammatory stimulation. The same is true for TGFβ1: a significant lower expression is detected for the PM (37.27% ± 1.82% to 54.94% ± 3.93%), early P (43.05% ± 1.31% to 92.84% ± 8.99%) and late P (41.68% ± 6.59% to 79.14% ± 9.27%) condition after inflammatory stimulation. For HGF and PTGS2, only a significant upregulation is observed for the PM (4.13% ± 0.73% to 1.50% ± 0.35% and 44.05% ± 13.00 to 1.79% ± 0.44%, respectively) condition upon inflammatory stimulation.

### 2.7. Differential Expression and Regulation of TLR in MSCs

**Figure 6:**
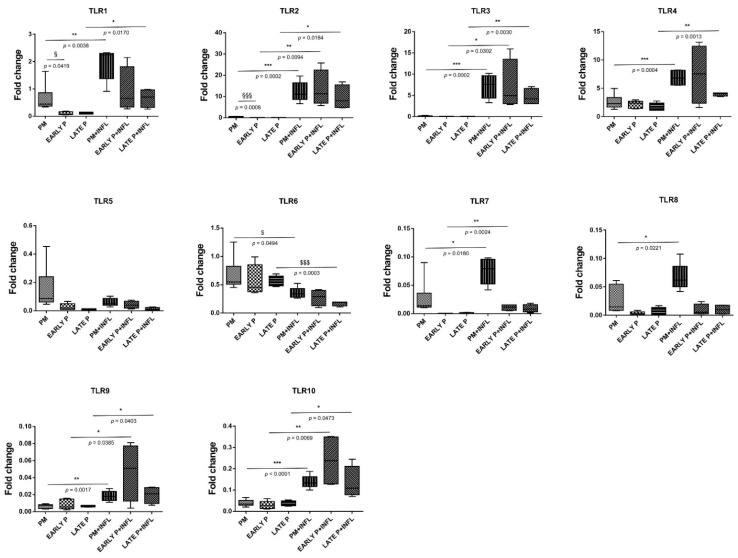
Distinct expression of Toll-like receptor (TLRs) and differential regulation in MSCs. The gene expression level of each cytokine is determined by qPCR analysis from basic MSCs and inflamed MSCs (INFL) during the primo culture (PM), early (EARLY) and late (LATE) passage (P). The values are expressed as mean ± SEM compared to the expression of the housekeeping gene (ACTB and GAPDH). *, **, *** Significant increase of expression of inflamed MSCs (INFL) versus basic MSCs (*p*-value: *p* ≤ 0.05, *p* ≤ 0.01, *p* ≤ 0.001, respectively). ^$, $$$^ Significant decrease of expression of inflamed MSCs (INFL) versus basic MSCs (*p*-value: *p* ≤ 0.05, *p* ≤ 0.001, respectively). ^§, §§§^ Significantly higher expression compared to the consecutive passage (*p*-value: *p* ≤ 0.05, *p* ≤ 0.001, respectively).

The expression of TLR in AT-MSCs is constitutively different and differentially regulated by both inflammation and cell-expansion (Figure 6). MSCs constitutively express 4 at low levels, whilst the expression of TLR1-3 and TLR5-10 was barely detected. After inflammatory stimulation, the expression of TLR1-10 is overall significantly higher, with exception of TLR5 and TLR6. The expression of TLR 1, 2, 3, 4, 9 and 10 is increased, whereas TLR 5 and 6 are downregulated. More specifically, for TLR2, TLR3, TLR9 and TLR10, a significantly higher expression is observed in the PM (28,3-fold; 56.1-fold; 2.8-fold and 3.6-fold, respectively), the early (686.4-fold; 151.8-fold; 5.6-fold and 8.5-fold, respectively) and the late passage (224.7-fold; 79.2-fold; 3.0-fold and 3.7-fold, respectively) condition after inflammatory stimulation compared to the non-inflammatory group. The expression of TLR1 and TLR4 in MSCs is significantly upregulated in the PM (3.0-fold and 2.6-fold, respectively) and the late passage (6.1-fold and 2.5-fold, respectively) condition after inflammatory stimulation. For TLR6 on the other hand, a significantly lower expression is observed for the PM (0.5-fold) and the late passage (0.3-fold) condition upon inflammatory stimulation. On the other hand, both conditions induced a mixed gene expression profile for TLR 7 and 8. MSCs show a significantly higher expression of TLR7 after exposure to inflammatory stimulation for the PM (2.9-fold) and early passage (66.9-fold) condition, whilst the expression of TLR8 is a little, but still significantly upregulated in the PM (2.5-fold) condition. Although the expression of TLR7–TLR10 is significantly upregulated upon inflammatory stimulation, it should be noted that their expression is almost not detected. No difference in expression is observed for TLR5 in the non-inflammatory and inflammatory group. Further, the cell-passaging is likely to not influence the expression of all these TLRs, with exception of TLR1 and TLR2. More specifically, no significant differences are observed between the PM condition, the early (P1–P2) passage condition and the late (P3–P4) passage condition. TLR1 and TLR2 are significantly higher expressed in the PM condition compared to the early passage condition (6.4-fold and 21.9-fold, respectively). Protein–protein interaction (PPI) network analysis was constructed and dissected for the identified TLRs. This part of the results was inserted within the discussion.

## 3. Discussion

Adipose tissue is considered an interesting alternative source of therapeutic MSCs for immunomodulation and regenerative medicine [15,16]. Increasing our knowledge regarding the influence of the tissue environment on the immuno-biology of AT-MSCs will contribute to the development of novel therapeutic approaches [17,18]. AT-MSCs in culture demonstrated a fibroblastic-like shape with a high capacity to adhere to plastic. The expansion of MSCs by cell-passaging allowed to increase their number without any significant sign of senescence. In general, AT-MSCs are relatively stable over long-term culture, maintaining a consistent expansion rate and exhibiting low levels of senescence [19]. Senescence may alter some features of MSCs, in particular at high passage level or in presence of cellular stress and damage [20]. Despite AT-MSCs differentiated in vitro into in osteoblasts and adipocytes, their beneficial effect seems to come from their immunomodulation and trophic ability rather than their multilineage potential [21]. The phenotype of AT-MSCs (Figure 2) presented the stromal surface markers CD105, CD90 and CD73 [22]. They lack expression of hematopoietic cell lineage markers CD45, CD14 and CD19. HLA-Dr considered as marker for antigen presenting cells (APCs) was not expressed. According to the ISCT, this phenotype is essential to define MSCs [7]. The absence of CD45, HLA-Dr, CD14 and CD19 expression is used to exclude contamination of cell-cultures by pan-leukocytes, APCs, monocytes/macrophages and B cells, respectively. Thus, AT-MSCs are not immunogenic and could not induce T-lymphocyte activation which is essential for preventing immune complication and graft rejection [23,24]. The expression of CD105 is reported to vary according to culture conditions and some findings indicate that distinct expression of CD105 might be related to specific multilineage and immunomodulatory potentials [25,26]. Considered as an endothelial marker, the expression of CD34 rapidly decreases during the cell-expansion. The variation some surface markers between freshly isolated cells and cells in culture is reported to be dependent on the tissue source of MSCs [27,28]. MSCs from adipose tissue are being considered CD34+ in situ and may represent a small proportion of the total cell population indicating a distinct subset of cells with enhanced progenitor activity [29,30,31,32]. The success of the reparative strategy based on the use of MSCs might be hampered by the harsh environment of injured tissue that is generally associated with inflammation/immunology reactions [33,34]. Wound healing is a complex process involving inflammatory, proliferative and reparative events [1,35,36]. Such process is mediated in large part by interacting molecular signals, primarily cytokines, chemokines, TLR and regulatory mediators that may greatly influence the recruitment, proliferation and engraftment of progenitor cells to expand the pool of therapeutic cells [37,38]. Consequently, it is essential to determine the transcriptional profile of these immunomodulatory and trophic mediators under challenging conditions [10].

We found that MSCs constitutively presented a distinct profile for each pattern of cytokine and chemokine. In addition to displaying different levels of expression, MSCs differentially and to some extent specifically modulated them in response to inflammation and cell-passaging. The cytokines IL-6, IL-1 Ra, IL-1β and TNFα as well as the chemokine IL-8 and CCL5 were constitutively presented by MSCs with different levels of expression. Moreover, we found that the expression of IL-6, IL-8, TNFα and CCL5 is downregulated upon cell passaging. This change in the cytokine profile suggests that MSCs are less interacting and soliciting the immune system during the proliferative phase of regeneration. On the other hand, the inflammatory setting differentially altered the cytokine profile of MSCs. A significant increase of IL-6, IL-8, IL-1β, IL-1Ra, TNFα and CCL5 expression is reached. It is likely that MSCs respond by promoting a pro-inflammatory environment suitable for tissue repair. Both IL-1 and TNF-α are always present during wound repair, but their pleiotropic and synergistic effects are not well understood. Moreover, it is suggested that TNF-α plays a role in wound healing process by promoting the recruitment and differentiation of tissue stromal cells [39]. In line with our observations, altered levels of TNF-α, IL-1 and IL-6 appear to be regulated differently in the early versus later chronic phases of wound healing, with overexpression dominating the later phases [40]. In parallel, recent results showed that, early in obesity and before inflammation was detected, high-fat (HF) diet durably and differently activated adipose stem cells (ASC) from the subcutaneous (SAT) and the visceral adipose tissue (VAT). Subcutaneous ASC from HF-fed mice strongly inhibited the proliferation of activated T lymphocytes, whereas visceral ASC selectively inhibited TNFα expression by macrophages and simultaneously released higher concentrations of IL6 [34]. These depot specific differences may contribute to the low-grade inflammation that develops with obesity in VAT while inflammation in SAT is delayed. It was reported that IL-6 is involved in the suppression of T-cell proliferation and local inflammation. This is an important finding, since we showed that MSCs significantly higher express IL-6 upon inflammation, which can reduce the rejection of transplanted allogeneic MSCs and enhance their capacity for tissue repair and regeneration [41]. A high level of chemokines and inflammatory cytokines, including CCL2, IL-1β and TNFα, allow an accumulation of M1 macrophages that further increased TNFα production to activate stem cells. Moreover, IL-1α, IL-13, IFNγ and TNFα cytokines may promote stem cell expansion and thus replenish their endogenous pool. These findings illustrate a cross-talk between stem cells and immune responses that determines the function and fate of stem cells in the process of tissue regeneration [42]. In agreement, we have shown that MSCs within an inflammatory environment increase the recruitment of T-cells by increasing IL-8 and CCL5 secretion. Following their migration, MSCs can impair lymphocyte proliferation and activation depending on their origin [43]. Reduced activation of inflammasome and suppressed production of IL-1β in macrophages were mainly responsible for beneficial effects of MSCs in many injured tissue model [44]. Upregulation of IL-1Ra will also favor allograft integration by blocking IL-1β-mediated cellular changes by competitive binding on their receptor [45]. Enhanced release of chemokines (CCL5, IL-8) and cytokines (TNF-a, IL-1 and IL-6) may modulate the features of neutrophil and macrophage known to cooperate during tissue repair [46]. The modulation of these cytokines and chemokines may be considered as a novel therapeutic approach in regenerative medicine [47].

We also evaluated the expression of regulatory mediators that are important for the control of wound healing process. MSCs constitutively presented different levels of expression HGF, HMOX1, IGFBP3, LIF, PTGS, PTGS2 and TGFβ1. We noted that neither the cell-passaging nor the inflammatory setting have modulated the expression profile of HLA-G, IGFBPs and LIF. We can speculate that these mediators, in a tissue-context dependent manner, are expressed and my participate to the tissue repair process driven by MSCs. Interestingly, TGF-β1 demonstrated a significant decrease upon inflammation despite an initial at high level. It seems that TGF-β1 is necessary for tissue repair at the earliest stages, although the molecule has inflammatory or anti-inflammatory effects depending on the cell type involved as well as the tissue context [38]. In contrast, AT-MSCs constitutively expressed low levels of HGF that was significantly increased under inflammatory setting regardless of the cell-passaging. HGF is an important factor for tissue repair by stimulating the proliferation and migration of respective progenitor cells [48,49]. The increase of HGF can also promote a M2 macrophage shift and regulatory T-cell generation that facilitates tissue regeneration [50]. Regardless of inflammatory setting, AT-MSCs constitutively maintained high levels of HO-1 with significant expression starting in PM condition. HO-1 is a highly inducible enzyme with cytoprotective, antioxidant and immunoregulatory properties that participate to tissue protective and adaptive responses [51]. In contrast to Mougiakakos D et al., we have not observed that inflammatory licensing of MSCs substantially altered HO-1 expression that compromise their immunomodulatory functions [52]. Of note, HO-1 was upregulated in BM-MSCs with a concomitant increase in the secretion of IL-1RA confirming that HO-1 couples activation of mitochondrial biogenesis to anti-inflammatory cytokine expression [53]. As previously documented, the expression and modulation of HO-1 by MSCs is dependent on cell-conditions and HO-1 is not mandatory for inducing a tolerogenic response [54]. In parallel, the expression of IDO1 and IDO2, two important immunoregulatory enzymes, was significantly upregulated by inflammation during PM and early culture condition. Such increase should be correlated with the fact that under inflammatory setting IDO are stimulated by MSCs [55]. A sustained IDO expression may thus create a local immune privilege that protects tissues from damage and allows tissues to heal [56]. The opposite effect was reported for the enzymes involved in the production of prostaglandin (PG) E2. PTGS1 (cyclo-oxygenase 1, COX1) and PTGS2 (COX2) were differentially modulated by inflammation with PTGS1 being significantly decreased, whereas PTGS2 was upregulated. To avoid exacerbation of tissue injury, regulatory pathways (IDO, HO-1, PGE2) are activated and allows to dampen the inflammatory response. At sites of injury, PGE2 production may promote the polarization of neutrophil and macrophage towards a tolerogenic phenotype appropriate for tissue repair and regeneration [57,58].

**Figure 7:**
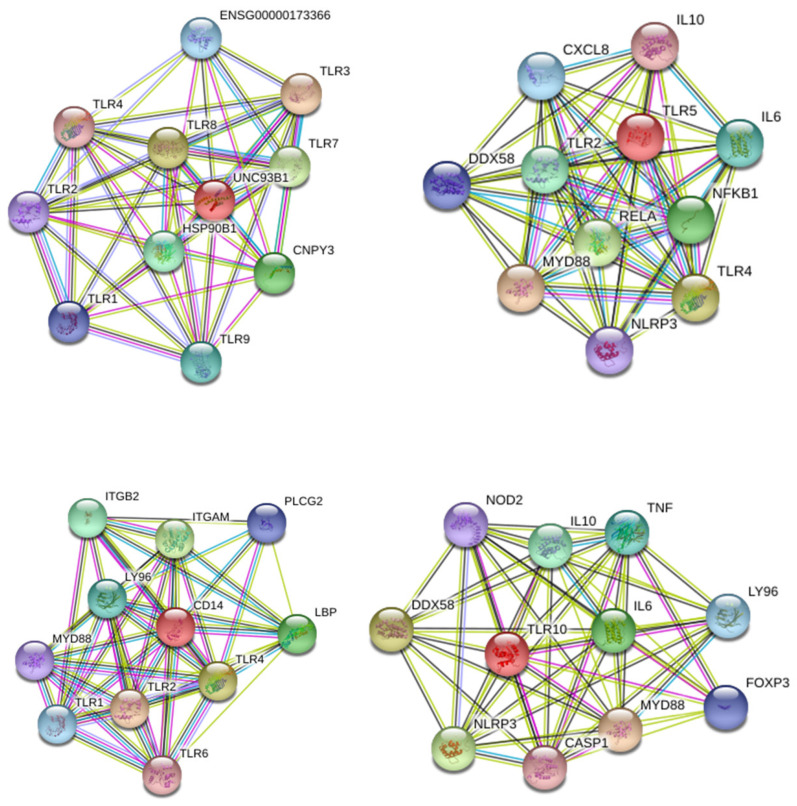
STRING 11.0 analysis of known and predicted TLR-protein interactions. The interactions include direct (physical) and indirect (functional) associations. Network nodes represent proteins and edges represent protein-protein associations (specific and meaningful). The lines represent the existence of the several types of evidence used in predicting the associations (high confidence score 0.9). The interactions are shown in different colors: black is co-expression; dark blue is co-occurrence; purple is experimental evidence; light green is text mining.

**Figure 8:**
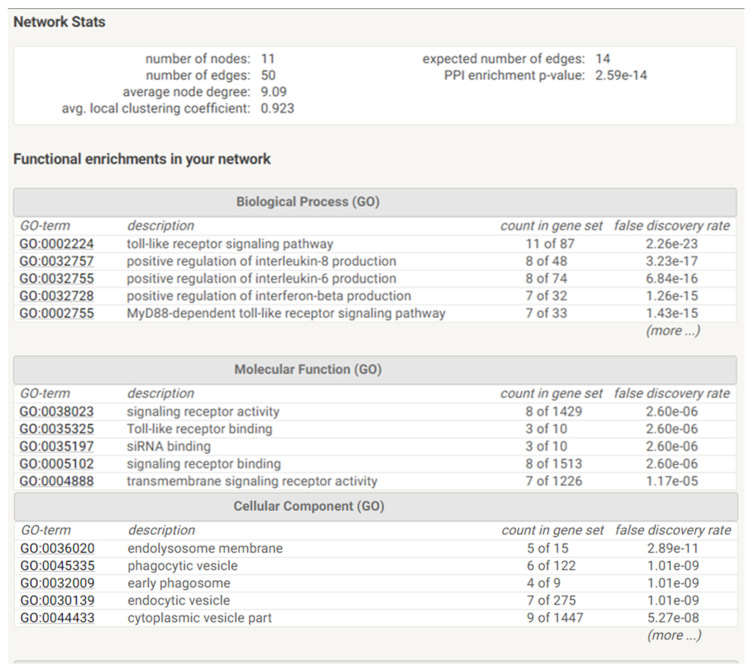
Functional enrichments and Statistics of TLR networks interactions and association. Gene Ontology (GO) is used to perform enrichment analysis for biological process, molecular functions and cellular components.

We then observed that several TLRs were differentially expressed by AT-MSCs and significantly modulated by both cell-passaging and inflammation. The significant modifications documented for TLR expression will be discussed according to the importance and degree of the changes. Transplanted cells such as MSCs are often exposed, within injured tissues, to unfavorable conditions. TLRs acting as sentinels sense “danger signals” which, in turn, may affect the tissue repair process [59,60]. TLR are thus, instrumental in coordinating tissue repair and regeneration in a time and expression dependent manner [61]. The literature indicates that the source of MSCs as well as culture conditions may influence the expression profile of TLR [60]. As a comparison, dental pulp stem/progenitor cells (DPSCs) basically expressed high levels of TLR. In an inflamed environment, it upregulated TLR2, TLR3, TLR4, TLR5 and TLR8; downregulated TLR1, TLR7, TLR9 and TLR10; and abolished TLR6 expression [62]. TLRs triggers expression of genes mainly linked to innate immune responses through the utilization of specific or common signaling pathways. They are finely regulated by TIR (toll/IL-1 receptor) domain-containing adaptors, such as MyD88, TIRAP/Mal, TRIF and TRAM [63]. It has been reported that under in vitro hypoxic conditions, the expression of specific TLR is affected [64]. As TLR2 was significantly upregulated during cell-expansion in the presence of inflammation, it may promote the proliferation of MSCs and the production of vascular endothelial growth factor (VEGF) IL-6, IL-8 and TNFα during which are required in the early phases of tissue regeneration injury [65,66,67]. On the other hand, TLR3 and TLR 4 have been described to be involved in tissue repair according to several experimental settings [68]. A substantial augmentation of both TLR expression is reached under inflammation setting and may result in the increase of IL-6 and IL-8 expression, which allows MSCs to induce M2 type monocytes polarization that play important roles during tissue injury repair [69,70] [71]. Moreover, MyD88 was shown to regulate the proliferation and differentiation of AT-MSCs [72,73]. In turn, the expression of TLR5, TLR6, TLR7, TLR8, TLR9 and TLR10 was low and not altered by cell-expansion and inflammation except for TLR5 (increase) and TLR6 (decrease). MSCs from umbilical cord blood [74] and amnion [75], showed increase of cytokines and chemokines associated with their TLR5 and TLR6 expression. This increase may promote the migration of MSCs from adipose tissue to target tissues [76] as well as favor a tolerogenic environment suitable for tissue regeneration [77]. Finally, as differential utilization of TIR domain-containing adaptors may provide specificity of individual TLR-mediated signaling pathways, we aimed to have a view on the proteins and functional interactions that may occur. Using STRING v11 [78], we visualized TLR interaction networks (Figure 7) and performed gene-set enrichment analysis (Figure 8). TLR may establish several networks, interactions and associations with different proteins. Functional enrichment analysis showed that several biological process, molecular functions and cellular components linked to TLRs are involved in inflammatory and immune response. Decrypting how all of these pathways act in concert with one another will help to better define the regulatory network by which AT-MSCs induce the repair of the tissue environment.

## 4. Materials and methods

### 4.1. Adipose Tissue Collection 

Human adipose tissue (waste material) is collected after obtaining informed consent from donors undergoing elective liposuction in cooperation with the Department of Plastic Surgery of the UZ-Brussels (Brussels, Belgium) and the ATLAS clinic (Brussels, Belgium).

### 4.2. Isolation and Culture of MSCs

The isolation and culture procedure can be briefly explained as follows: 125 mL of liposuction material is extensively washed (centrifugation 3 min at 600× *g*) with equal volumes of phosphate-buffered saline (PBS) (Sigma-Aldrich, Diegem, Belgium) to remove erythrocytes. The adipose tissue material is incubated for 45 min at 37 °C with dissociation buffer (1:1). This solution is composed of 1 % (*v*/*v*) bovine serum albumin (BSA) (Sigma-Aldrich, Diegem, Belgium) and 1 mg/mL collagenase A (Roche Applied Science, Vilvoorde, Belgium) dissolved in PBS. After this digestion step, the sample is passed through a mesh filter to remove connective tissue debris. Subsequently, the filtrate is washed by centrifugation (10 min 4 °C at 600× *g*) and the supernatant removed. The recovered pellet is suspended in 50 mL PBS supplemented with 1 % (*v*/*v*) BSA and centrifuged again for 10 min at 600× *g* (4 °C). The new pellet is resuspended in 30 mL of PBS, supplemented with 1% (*v*/*v*) BSA. The resulting cell suspension is carefully brought on top of 15 mL of Ficoll gradient solution (Sigma-Aldrich, Diegem, Belgium) and centrifuged for 20 min at 1000× *g* (4 °C). Upon centrifugation, the upper layer is removed and the cell interface layer is carefully collected in 50 mL PBS supplemented with 1% (*v*/*v*) BSA. The cell suspension is centrifuged for 10 min at 600× *g* (8 °C) and the supernatant is removed. The cell pellet is cultured in a 58 cm^2^ petri dish (Greiner Bio One, Vilvoorde, Belgium) in Dulbecco’s Modified Eagle Medium (Lonza, Braine-1′Alleud, Belgium) with Low-Glucose, l-glutamine, Sodium Pyrovate, Phenol Red but no HEPES. This medium was then supplemented with 10% (*v*/*v*) fetal bovine serum (HyClone, Perbio Science, Erembodegem-Aalst, Belgium), 7.33 IU/mL benzyl penicillin (Continental Pharma, Brussels, Belgium), 50 μg/mL streptomycin sulphate (Sigma-Aldrich, Diegem, Belgium), 2.5 μg/mL Fungizone (Invitrogen, Merelbeke, Belgium). After 5 days of culture at 37 °C, under an atmosphere of 5% CO_2_ and 95% air, non-adherent cells are removed by replacing the medium. During the culture period, the medium is changed once a week. When the culture reaches sub-confluence (80%), the cells are harvested using TrypLE Select (Sigma-Aldrich, Diegem, Belgium) and counted using a 0.4 % (*w*/*v*) trypan blue dye solution (Sigma-Aldrich, Diegem, Belgium). For further cell-expansion, cells are replated at a low-density of 1000 cells/cm^2^ followed by passage every time those cells reached sub-confluence. Primo-culture (PM), early-passage (passage 1∼2) and late-passage (passage 3∼4) of MSCs were evaluated. The morphology of MSCs is observed by light microscopy.

### 4.3. The Culture Characteristics of Adipose MSCs

During their expansion (from PM until P4), the number of MSCs generated when the cultures reached the sub-confluency, was evaluated as a function of culture time by Trypan blue exclusion assay (Lonza, Braine-1′Alleud, Belgium). Therefore, the cumulative cell number at each passage was calculated as a ratio of total number of cells harvested to total number of cells seeded multiplied by the total number of cells from the previous passage. 

During their expansion (from PM until P4), MSCs were assessed for their expression of SA β-Galactosidase (SA-ß-Gal) which is a common marker of senescence. MSCs cells were plated in a 48 well-plate for 24 h before staining cells with the Senescence Detection Kit (BioVision, Milpitas, CA, USA). SA-ß-Gal catalyzes the hydrolysis of X-gal, which produces a blue color in senescent cells. The number of blue MSCs out of 100 total cells was therefore scored using an inverted microscope. The results were presented as the mean percentage (±SEM) of SA β-Gal positive cells compared to total counted cells.

### 4.4. Phenotype of MSCs

The cells isolated from the adipose cultures are characterized for the expression of a panel of markers (Table 1). This screening is performed by flow cytometry using fluorochrome labeled monoclonal antibodies.

### 4.5. In Vitro Lineage Differentiation of MSCs

The cells isolated from the adipose cultures are investigated for their lineage differentiation capacities.

#### 4.5.1. Osteogenesis 

Cells recovered from the cultures, were cultivated in osteogenesis differentiation medium consisting of 90% (*v*/*v*) STEMPRO^®^ Osteocyte/Chondrocyte Differentiation Basal Medium and 10% (*v*/*v*) STEMPRO^®^ Osteogenesis Supplement (all from Gibco, Life Technologies, Merelbeke, Belgium) for 3 weeks. The medium was changed every 2 days. To confirm the osteogenic differentiation, cells were fixed with 4% (*w*/*v*) paraformaldehyde (PFA) during 30 min at room temperature (RT). After fixation, the wells were rinsed twice with distilled water. Next, the cells were stained with 2% (*w*/*v*) Alizarin Red S (pH 4.2) solution (Sigma, Bornem, Belgium) for 30 min at RT. Finally, wells were rinsed three times with distilled water and visualized with a phase contrast microscope (Nikon).

#### 4.5.2. Adipogenesis

Cells recovered from the cultures, were cultivated in adipogenesis differentiation medium consisting of 90% (*v*/*v*) STEMPRO^®^ Adipocyte Differentiation Basal Medium and 10 % (*v*/*v*) STEMPRO^®^ Adipogenesis Supplement (all from Gibco, Life Technologies, Merelbeke, Belgium) for 2 weeks. The medium was changed every 2 days. To confirm the adipogenic differentiation, cells were fixed with 4% (*w*/*v*) PFA during 30 min at RT. Following fixation, the wells were rinsed twice with PBS and the cells stained with 0.5% (*v*/*v*) Oil Red O in isopropanol (Sigma, Bornem, Belgium) for 30 min at RT. Finally, wells were rinsed three times with distilled water and visualized with a phase contrast microscope (Nikon).

### 4.6. Inflammatory Stimulation 

MSCs were incubated for 18 h with a pro-inflammatory cytokine cocktail. The cocktail used to stimulate the cells is composed of four cytokines including 25 ng/mL interleukin (IL)-1β (Peprotech, Rocky Hill, NJ, USA), 50 ng/mL tumor necrosis factor (TNF)-α, 10 ng/mL interferon (IFN)-α and 50 ng/mL IFN-γ (all from Prospec Inc., Rehovot, Israel). This cocktail of cytokines was used in each experiment requiring an inflammatory stimulation.

### 4.7. Transcriptional Profile of MSCs 

The transcriptional profile of TLR, cytokine and regulatory mediators by MSCs was determined by quantitative polymerase chain reaction (qPCR) as previously performed [79,80,81]. Total mRNA was extracted using the TriPure Isolation Reagent^TM^ (Roche Applied Science) and quantified at 260 nm using a Nanodrop^TM^ spectrophotometer (Thermo Scientific, Waltham, MA, USA). Total RNA was reverse transcribed into cDNA using iScript^TM^ cDNA Synthesis Kit (BioRad, Nazareth, Belgium) followed by cDNA purification with the GenElute PCR clean up kit (Sigma–Aldrich) according to the manufacturer’s instructions. cDNA products were used for quantitative amplification of the target genes. The gene expression assays used in this study are listed in Table 2. All samples were done in duplicate and each run included two no template controls and a serial dilution of a pooled cDNA mix from all samples to estimate the qPCR efficiency. The qPCR reaction mix consisted of 10 µL TaqMan Fast Advanced Master Mix (Life Technologies), 1 µL 20× Assay-on-Demand Mix (Life Technologies) and 2 µL of cDNA in a 20 µL volume adjusted with DNase/RNase-free water. qPCR conditions, using the StepOne Plus system (Life Technologies), were as follows: incubation for 20 s at 95 °C, followed by 40 cycles of 1 s denaturation at 95 °C, annealing for 20 s at 60 °C (Life Technologies). qPCR efficiency was estimated by the StepOne Plus System’s Software and the data were only used when the calculated PCR efficiency ranged from 0.85–1.15. Moreover, for selecting reliable reference genes to normalize the qPCR data, we first evaluated the expression stability of five candidate reference genes: glyceraldehyde 3-phosphate dehydrogenase (GAPDH), beta-2-microglobulin (B2M), hydroxy-methylbilane synthase (HMBS), beta-actin (ACTB) and ubiquitin C (UBC). According to geNorm^®^ (Biogazelle, Gent, Belgium), the optimal number of reference targets to be used in this experiment was 2 (V < 0.15). As such, ACTB and GAPDH were selected as the most stable reference genes in all samples using qbasePLUS^®^ software (geNorm^®^). Relative mRNA expression levels of the target genes in AT-MSCs were thus normalized against the geometric means of both reference gene mRNAs.

### 4.8. Construction and Analysis of the Protein–Protein Interaction Network 

The PPI network was predicted using the Search Tool for the Retrieval of Interacting Genes (STRING) online data-base (http://string-db.org; version 11.0) (Accessed on 10 March 2021). The STRING database (relies on many data sources) was used also for the functional annotation and pathway enrichment analysis.

### 4.9. Statistical Analysis

The results are expressed as the mean ± standard error of the mean (SEM) from 7 different donor samples. Each sample for each condition was analyzed in triplicate. For statistical comparison, the unpaired *t*-test is performed. A *p*-value less than 0.05 is considered statistically significant (Prism, Graph-Pad Software, La Jolla, CA, USA).

## 5. Conclusions

AT-MSCs promote immunomodulation and tissue repair through different signaling pathways. They act by sensing the tissue environment as well as by modulating the features of local immune and progenitor cells. The current study describes, for the first time, the impact of the combination of inflammation and cell-expansion (two critical signals within the tissue site) on the sensitivity and responsiveness of AT-MSCs by exploring the transcriptional characteristics of some trophic and regulatory gene. AT-MSCs maintained a long-term expansion level in our cell culture setting, without significant sign of senescence. Senescence and viability should be evaluated during the expansion of AT-MSCs to guarantee the quality of the preparation. Regarding the therapeutic profile of AT-MSCs, several cytokines, chemokines, regulatory factors and TLRs, described as important for their immuno-reparative potential, were differentially expressed and modulated. Such observations are encouraging and have to be extended to further define these gene differences on a protein level. Setting up such an assay may be useful as a distinct protein profile can be obtained. Inflammation may thus increase the sensitivity and responsiveness of MSCs to their surroundings by at least three means (i) upregulating their pattern of TLRs that act as sentinels of infection and injury/damage; (ii) inducing a shift in their cytokine profile which contribute to the homing and activation of immune cells with a pro-reparative and anti-inflammatory phenotype; (iii) triggering the expression of regulatory and protective mediators to guarantee tissue homeostasis. Depending on their expression, modulation and role, these cytokines, chemokine, regulatory mediators and TLR may represent appropriate targets (on/off switch strategy to increase or decrease a pathway) to improve the therapeutic efficiency of AT-MSCs or a relevant set of biomarkers that indicate the potential efficiency of AT-MSCs. A defined transcriptomic signature would support the identification of sub-populations of AT-MSCs with a specific immuno-trophic profile suitable for immunomodulation and tissue repair. Such observations may strength our knowledge about the influence of tissue surrounding signals such as inflammation and proliferation that may improve or hamper the tissue healing process of MSCs. However, we have to keep in mind that the in vivo condition might be different as these findings are obtained from in vitro experiments. It is important to point out that most biological properties of MSCs are derived from studies with cells expanded ex vivo, rather than in vivo research. For the in vivo investigation of MSCs, different animal models are widely used, but this is not sufficient to properly reflect what is happening in the human body. It is conceivable that in a near future, the details about the in vivo interplay between AT-MSCs and their environment as well as their collective actions during tissue repair, will accelerate the progress in the field regenerative medicine. Hence, feasible approaches are needed for monitoring the inflammatory and immunological status of patients at the time therapeutic cells are infused to help optimize cell-based therapy. Indeed, further investigations in the effects of inflammatory factors, which fluctuate considerably in the microenvironment of a tissue lesion, on the immuno-biology of stem cells will accelerate the development of novel therapeutic approaches. Collectively, these discussions, reflections and perspectives should increase the knowledge regarding the therapeutic value of AT-MSCs and hopefully be clinically relevant.

## Figures and Tables

**Table 1:** List of Antibodies.

Primary Antibody	Company	Species	Dilution
CD34-PC5	BD	mouse	1/20
CD73-PE	BD	mouse	1/20
CD90-APC	BL	mouse	1/20
CD105-FITC	AC	mouse	1/20
CD45-PE	BD	mouse	1/20
CD14-PE	BD	mouse	1/20
CD19-PE	BD	mouse	1/20
HLA-DR-PerCP	BD	mouse	1/20

**Table 2:** Taqman Gene Expression Assays.

Genes	Assay-on-Demand ID	Amplicon Length (bp)	Supplier
**Housekeeping**			
GAPDH	Hs99999905_m1	122	AB
B2M	Hs99999907_m1	75	AB
HMBS	Hs00609296_g1	69	AB
ACTB	Hs99999903_m1	171	AB
UBC	Hs00824723_m1	71	AB
**Cytokines**			
IL-6	Hs00174131_m1	95	AB
IL-8	Hs00174103_m1	101	AB
IL-1b	Hs01555410_m1	91	AB
CCL5	Hs00982282_m1	70	AB
IL-1Ra	Hs00991010_m1	97	AB
TNF-α	Hs00174128_m1	80	AB
**TLR**			
TLR-1	Hs00413978_m1	72	AB
TLR-2	Hs02621280_s1	112	AB
TLR-3	Hs01551079_g1	144	AB
TLR-4	Hs00152939_m1	89	AB
TLR-5	Hs01920773_s1	89	AB
TLR-6	Hs01039989_s1	79	AB
TLR-7	Hs01933259_s1	121	AB
TLR-8	Hs00152972_m1	89	AB
TLR-9	Hs00370913_s1	70	AB
TLR-10	Hs01935337_s1	153	AB
**Regulatory**			
HGF	Hs00300159_m1	92	AB
HLA-G	Hs00365950_g1	91	AB
HMOX1	Hs01110250_m1	82	AB
IDO1	Hs00984148_m1	66	AB
IDO2	Hs01589373_m1	101	AB
IGFBP2	Hs01040719_m1	54	AB
IGFBP3	Hs00181211_m1	78	AB
LIF	Hs00171455_m1	66	AB
PTGS1	Hs00377726_m1	60	AB
PTGS2	Hs00153133_m1	75	AB
TGFB1	Hs00998133_m1	57	AB

AB: Applied Biosystems.

## Data Availability

The data presented in this study might be available depending on the type of the demand and the use and are linked to the authorities’ authorization. A request must be sent to the corresponding author with the permission of all authors.

## Abbreviations

CCL	chemokine ligand
HGF	hepatocyte growth factor
HLA	human leukocyte antigen
HMOX	heme oxygenase
IDO	indoleamine 2.3-dioxygenase
IGFBP	insulin-like growth factor binding-protein
IL	interleukin
LIF	leukemia inhibitory factor
MSCs	mesenchymal stromal cells
P	passage
PM	primo culture
PTGS	prostaglandin endoperoxide synthase
SEM	standard error of the mean
TGF	transforming growth factor
TLR	Toll-like receptor
TNF	tumor necrosis factor
